# Thoracoscopic Segmentectomy Within an Enhanced Recovery Pathway Improves Days Alive and Out of Hospital Compared with Lobectomy

**DOI:** 10.1093/icvts/ivag043

**Published:** 2026-02-06

**Authors:** Lin Huang, Henrik Kehlet, René Horsleben Petersen

**Affiliations:** Department of Cardiothoracic Surgery, Copenhagen University Hospital, Rigshospitalet, Copenhagen 2100, Denmark; Department of Thoracic Surgery, Shanghai Chest Hospital, Shanghai Jiao Tong University School of Medicine, Shanghai 200030, China; Section for Surgical Pathophysiology, Copenhagen University Hospital, Rigshospitalet, Copenhagen 2100, Denmark; Department of Cardiothoracic Surgery, Copenhagen University Hospital, Rigshospitalet, Copenhagen 2100, Denmark

**Keywords:** days alive and out of hospital, enhanced recovery after surgery, video-assisted thoracoscopic surgery, lobectomy, segmentectomy

## Abstract

**Objectives:**

This study aims to investigate the first 90 days alive and out of hospital (DAOH90) following enhanced recovery thoracoscopic segmentectomy versus lobectomy.

**Methods:**

A retrospective analysis for consecutive thoracoscopic segmentectomies and lobectomies for clinical stage IA1-2 non-small cell lung cancer (cIA1-2 NSCLC) was performed between January 2018 and March 2024. All factors contributing to reduced DAOH90 were analyzed individually. The association between surgical extent and DAOH90 was assessed using a multivariable logistic regression model. Sensitivity analyses were performed after propensity score matching.

**Results:**

Of 720 patients, 591 underwent lobectomy and 129 underwent segmentectomy. Compared with lobectomy, patients undergoing segmentectomy had poorer lung function and exercise capacity, more comorbidities, slightly longer operative duration, and less blood loss. The median DAOH90 was 1 day longer after segmentectomy than lobectomy (87 vs 86 days, *P* = .049). Air leak > 1 day (38.3% vs 40.0%), pneumonia (13.3% vs 18.3%), and pain (13.3% vs 23.3%) were important reasons to reduce DAOH90, all occurring more frequently in the lobectomy group. Social factors (37.5% vs 25.8%) were also a predominant cause in both groups, particularly after segmentectomy. Other causes were less common. In multivariable analysis, lobectomy (vs segmentectomy, OR 1.44, *P* = .048) was identified as an independent predictor of shorter DAOH90, along with body mass index, lung function, and cardiac comorbidity. The results of the sensitivity analysis were consistent with these findings.

**Conclusions:**

Following an enhanced recovery thoracoscopic protocol, segmentectomy for well-selected patients with cIA1-2 NSCLC may result in longer DAOH and less postoperative complications compared to lobectomy.

## Graphical abstract

**Figure:**
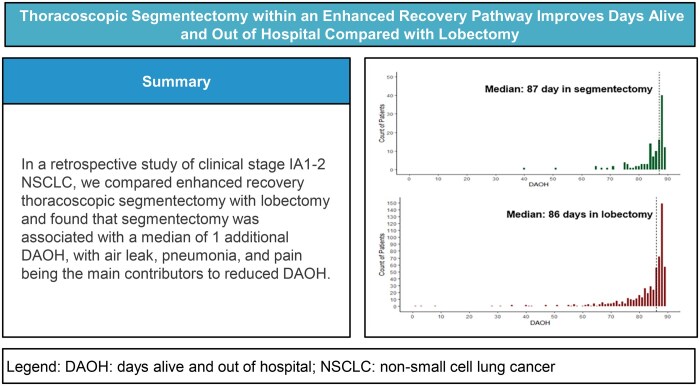

## INTRODUCTION

On the basis of the JCOG0802 and CALGB140503 trials,^1^^,^^2^ segmentectomy has been established and recommended as a standard surgical treatment for peripheral cIA1-2 non-small cell lung cancer (NSCLC).^3^ Secondary or post-hoc analyses of these trials suggested that perioperative outcomes were comparable between segmentectomy and lobectomy,^4^^,^^5^ while segmentectomy may preserve more lung function.^6^ However, perioperative outcomes specific to video-assisted thoracoscopic surgery (VATS) remain a matter of debate when comparing the 2 surgical types.^7–9^ Notably, none of the available evidence has addressed the implementation of enhanced recovery after surgery (ERAS) or “fast-track” surgery protocols. It remains unclear whether patients undergoing VATS within an ERAS protocol achieve similar perioperative outcomes after segmentectomy and lobectomy.

Furthermore, most existing studies have assessed perioperative outcomes using time-point measurements such as length of stay (LOS), morbidities, or mortality.^4^^,^^5^^,^^7–9^ However, these indicators may not adequately reflect the overall quality of perioperative care. Days alive and out of hospital (DAOH) provides a pragmatic, patient-centred outcome by integrating any hospitalization and mortality into a single metric. It is conceptually similar to survival assessment. Our previous studies have demonstrated that DAOH is a valuable metric for evaluating perioperative quality and that reduced DAOH within the first 90 days (DAOH90) is closely associated with surgery.^10^^,^^11^ Nonetheless, evidence regarding DAOH in the context of ERAS VATS segmentectomy remains lacking.

Therefore, this study aimed to evaluate DAOH90 after ERAS VATS segmentectomy and to identify risk factors for reduced DAOH90 in comparison with VATS lobectomy.

## METHODS

### Ethical statement

The study protocol was approved by the Danish Patient Safety Authority (R-24008608) and the Danish Data Protection Agency (P-2024-15514) prior to study initiation. The requirement for informed consent was waived in accordance with the Danish Committee System on Health Research Ethics.

### Study design and setting

This study was a retrospective analysis of prospectively collected data from consecutive patients who underwent VATS anatomical resection (segmentectomy or lobectomy) for NSCLC at the Department of Cardiothoracic Surgery, Copenhagen University Hospital, Rigshospitalet, Denmark, between January 2018 and March 2024. Segmentectomy rates remained increased over the study period. Perioperative care pathways, surgical procedures, criteria for chest drain removal and discharge, and post-discharge management followed previous publications.^10^^,^^11^ All data were obtained from the national electronic health record system, which ensures complete enrolment and follow-up as required for economic reimbursement. Data were accessed through the digital healthcare platform (Epic Systems, Madison, WI, United States) and subsequently recorded in the Research Electronic Data Capture tool (Vanderbilt University, Nashville, TN, United States). The study was reported in accordance with the *Strengthening the Reporting of Observational Studies in Epidemiology* guidelines.^12^

### Eligible patients

Inclusion criteria were: (1) age ≥ 18 years, (2) scheduled for VATS anatomical resection, and (3) residence in Eastern Denmark.

Exclusion criteria were: (1) clinical stage higher than IA2, (2) middle lobectomy or bi-lobectomy, and (3) anatomical lung resection within 90 days prior to the index surgery.

### Variables

Demographic variables included age, sex, body mass index, and smoking status. Preoperative clinical characteristics included percentage of predicted forced expiratory volume in 1 s (FEV_1_%pre), American Society of Anaesthesiologists (ASA) classification, comorbidities, Charlson Comorbidity Index (CCI), clinical stage, and location of lesion. Intraoperative variables included extent of resection, operative duration, and blood loss. Pathological characteristics comprised tumour histology. Postoperative outcomes included LOS, duration of chest drainage, postoperative complications, Clavien-Dindo classification, 90-day readmission, 90-day mortality, and DAOH90. All admissions to any hospital for any course were recorded and counted in DAOH.

The primary endpoint was the difference in DAOH90 between lobectomy and segmentectomy.


DAOH=90−(index LOS+readmitted LOS within     postoperative 90 days+the days until the day    of death before postoperative day (POD) 90)

Secondary end-points included the difference in reasons for reduced DAOH90 between the 2 groups, as well as the association of the 2 surgical approaches with inferior DAOH90 (defined as values lower than or equal to the median of DAOH90). All factors associated with index admission, readmission, and mortality were assessed individually as reasons.

Specific definitions of the study variables are provided in **Table S1**.

### Statistical analysis

The statistical procedure followed the corresponding guidelines.^13^ There was no missing data. Continuous variables were presented as median with interquartile range (IQR) due to non-normally distributed according to the Kolmogorov-Smirnov and Shapiro-Wilk tests. Categorical variables were presented as numbers and percentages. The Mann-Whitney *U*-test was used to compare continuous variables, while Fisher’s exact test or the chi-squared test was applied for categorical variables. Variables with *P* < .1 in the univariable logistic regression analysis, together with surgical extent, were included in the multivariable model to identify independent predictors. A sensitivity analysis was performed using propensity score matching (PSM) based on all preoperative characteristics. PSs were estimated with a multivariable logistic regression model, incorporating all preoperative variables. Matching was conducted using a 1:1 nearest-neighbour algorithm with a caliper width of 0.10 on the logit of the PS, without replacement. Covariate balance was assessed using standardized mean differences, with values < 0.10 indicating adequate balance. A 2-sided *P* < .05 was considered statistically significant. All statistical analyses and visualizations were conducted using R software (version 4.5.1; R Foundation for Statistical Computing, Vienna, Austria).

## RESULTS

**Figure 1.**
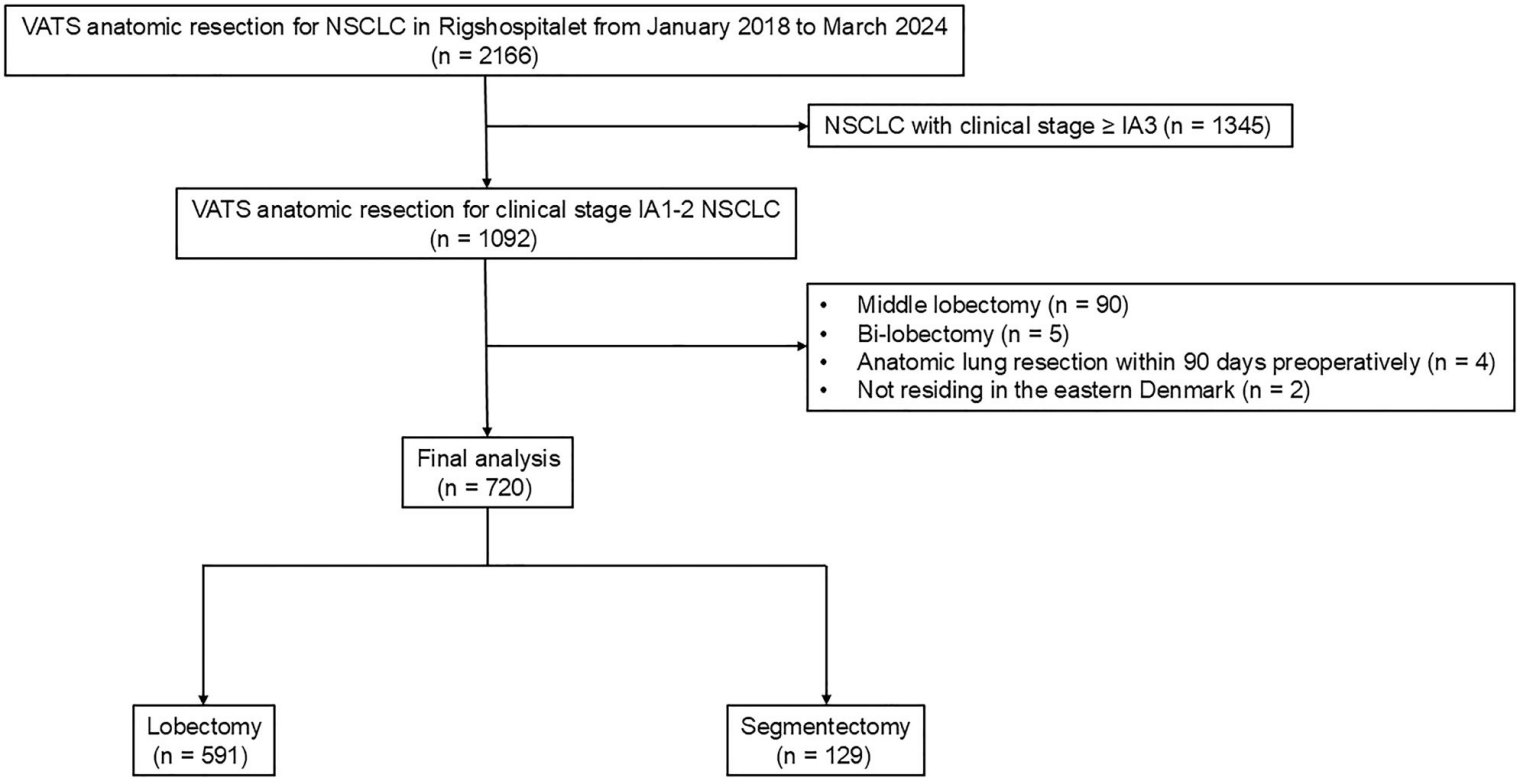
Patient Enrolment. NSCLC: non-small-cell-lung cancer, VATS: video-assisted thoracoscopic surgery.

Of 2166 patients who underwent VATS anatomical resection for NSCLC, 720 met the inclusion criteria and were analyzed (**Figure 1**). The cohort comprised 591 lobectomies and 129 segmentectomies. Patients in the segmentectomy group had a higher proportion of clinical stage IA1 disease compared with those in the lobectomy group (51.2% vs 21.8%, *P* < .001). No patient experienced conversion to open surgery or required residual tumour resection. All resections were R0.

Compared with lobectomy, patients undergoing segmentectomy had a median FEV_1_%pre approximately 10% lower, higher ASA classifications (III: +2%, IV: +4%), and a higher CCI. Tumour in the segmentectomy group were more frequently located in the left upper and right lower lobes. The distribution of resected segments is presented in **Table S2**. Segmentectomy required a median of 9 minutes longer operative time with less blood loss. Postoperatively, segmentectomy was associated with a median 1-day shorter LOS compared with lobectomy. In-hospital morbidity was 11.2% lower after segmentectomy. This difference was not observed during the period up to POD30 after discharge, but reappeared during POD31-90. Ninety-day readmission rates and mortality did not differ significantly between the 2 groups. Detailed perioperative characteristics are summarized in **Tables 1 and 2**, and **Tables S3 and S4**.

**Table 1.** Preoperative and Intraoperative Characteristics

Characteristic	**Overall** ^a^ *n* = 720	**Lobectomy** ^a^ *n* = 591	**Segmentectomy** ^a^ *n* = 129	SMD	***P*-value** ^b^
Age (years)	70 (64, 75)	70 (64, 75)	71 (66, 75)	−0.09	.325
Male	267 (37.1%)	219 (37.1%)	48 (37.2%)	−0.00	.974
BMI (kg/m^2^)	25 (22, 28)	25 (22, 28)	25 (22, 28)	0.01	.630
Smoke status				0.08	.290
Never smoking	85 (11.8%)	71 (12.0%)	14 (10.9%)		
Former smoker	391 (54.3%)	313 (53.0%)	78 (60.5%)		
Current smoker	244 (33.9%)	207 (35.0%)	37 (28.7%)		
FEV_1_%pre	86 (72, 100)	88 (74, 101)	78 (65, 93)	0.40	**<.001**
ASA				−0.24	**.011**
I-II	143 (19.9%)	124 (21.0%)	19 (14.7%)		
III	558 (77.5%)	456 (77.2%)	102 (79.1%)		
IV	19 (2.6%)	11 (1.9%)	8 (6.2%)		
Diabetes	79 (11.0%)	64 (10.8%)	15 (11.6%)	−0.03	.793
Hypertension	295 (41.0%)	240 (40.6%)	55 (42.6%)	−0.04	.672
Pulmonary comorbidity	179 (24.9%)	144 (24.4%)	35 (27.1%)	−0.06	.510
Cardiac comorbidity	136 (18.9%)	119 (20.1%)	17 (13.2%)	0.07	.067
Stroke	48 (6.7%)	35 (5.9%)	13 (10.1%)	−0.08	.086
CCI	2 (1, 3)	2 (0, 2)	2 (2, 3)	−0.49	**<.001**
Clinical stage				0.29	**<.001**
cIA1	195 (27.1%)	129 (21.8%)	66 (51.2%)		
cIA2	525 (72.9%)	462 (78.2%)	63 (48.8%)		
Location of lesion				0.38	**<.001**
LLL	105 (14.6%)	73 (12.4%)	32 (24.8%)		
LUL	196 (27.2%)	133 (22.5%)	63 (48.8%)		
RLL	135 (18.8%)	106 (17.9%)	29 (22.5%)		
RUL	284 (39.4%)	279 (47.2%)	5 (3.9%)		

Bold *P*-value indicates statistical significance.

a Median (interquartile range); *n* (%).

b Wilcoxon rank sum test; Pearson’s Chi-squared test; Fisher’s exact test.

Abbreviations: BMI: body mass index; CCI: Charlson Comorbidity Index; FEV1%pre: percentage of predicted forced expiratory volume in 1 second; LLL: left lower lobe; LUL: left upper lobe; PSM: propensity score matching; RLL: right lower lobe; RUL: right upper lobe; SMD: standardized mean difference.

**Table 2.** Postoperative Outcomes

Variables	**Overall** ^a^ *n* = 720	**Lobectomy** ^a^ *n* = 591	**Segmentectomy** ^a^ *n* = 129	ARF	95% CI	***P*-value** ^b^
Surgical duration (minutes)	99 (85, 116)	95 (80, 115)	104 (93, 119)	−7.87	−12.32 to −3.41	**<.001**
Blood loss (mL)	20 (10, 50)	20 (10, 50)	20 (10, 40)	18.29	6.82 to 29.77	**.040**
Length of stay (day)	3 (2, 6)	3 (2, 6)	2 (2, 4)	1.18	−0.04 to 2.41	**.041**
Duration of chest drainage (day)	1 (1, 3)	2 (1, 4)	1 (1, 2)	0.89	0.14 to 1.63	**.003**
Postoperative complications in the hospital						
Overall	301 (41.8%)	259 (43.8%)	42 (32.6%)	−11.27	−20.76 to −1.77	**.019**
Highest CDC 1	138 (19.2%)	119 (20.1%)	19 (14.7%)			
Highest CDC 2	86 (11.9%)	73 (12.4%)	13 (10.1%)			
Highest CDC 3	63 (8.8%)	53 (9.0%)	10 (7.8%)			
Highest CDC 4	10 (1.4%)	10 (1.7%)	0 (0.0%)			
Highest CDC 5	4 (0.6%)	4 (0.7%)	0 (0.0%)			
Postoperative complications during discharge to POD30						
Overall	93 (12.9%)	81 (13.7%)	12 (9.3%)	−4.40	−10.60 to 1.80	.177
Highest CDC 1	12 (1.7%)	11 (1.9%)	1 (0.8%)			
Highest CDC 2	30 (4.2%)	25 (4.2%)	5 (3.9%)			
Highest CDC 3	48 (6.7%)	43 (7.3%)	5 (3.9%)			
Highest CDC 4	2 (0.3%)	1 (0.2%)	1 (0.8%)			
Highest CDC 5	1 (0.1%)	1 (0.2%)	0 (0.0%)			
Postoperative complications during POD31 to POD90						
Overall	32 (4.4%)	31 (5.2%)	1 (0.8%)	−4.47	−7.29 to −1.65	**.026**
Highest CDC 1	8 (1.1%)	7 (1.2%)	1 (0.8%)			
Highest CDC 2	18 (2.5%)	18 (3.0%)	0 (0.0%)			
Highest CDC 3	5 (0.7%)	5 (0.8%)	0 (0.0%)			
Highest CDC 4	0 (0.0%)	0.0 (0.0%)	0 (0.0%)			
Highest CDC 5	1 (0.1%)	1 (0.2%)	0 (0.0%)			
90-days readmission	102.0 (14.2%)	89.0 (15.1%)	13.0 (10.1%)	−4.98	−11.40 to 1.43	.142
90-days mortality	10 (1.4%)	9 (1.5%)	1 (0.8%)	−0.75	−3.03 to 1.53	>.999
90-days alive and out of the hospital (day)	86 (81, 88)	86 (81, 88)	87 (84, 88)	−2.86	−4.53 to −1.20	**.049**

Bold *P*-value indicates statistical significance.

a *n* (%); median (interquartile range).

b Fisher’s exact test; Pearson’s Chi-squared test; Wilcoxon rank sum test.

Abbreviations: ARF: absolute risk difference; CDC: Clavin-Dindo classification; CI: confidence interval; POD: postoperative day.

**Figure 2.**
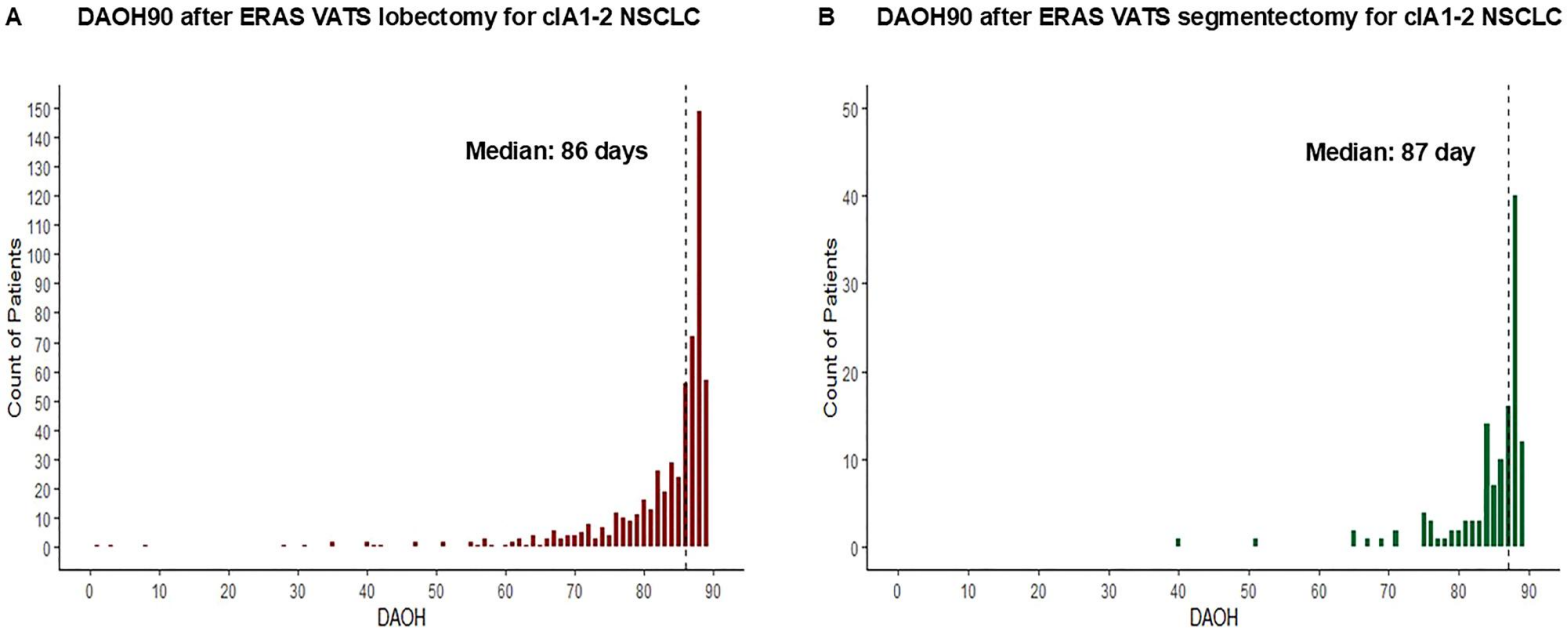
Distribution of the First 90 Days Alive and out of Hospital (DAOH90) after Enhanced Recovery Thoracoscopic (ERAS VATS) Lobectomy and Segmentectomy for Clinical Stage IA1-2 Non-Small Cell Lung Cancer (cIA1-2 NSCLC).

In the overall cohort, the median DAOH90 was 86 days (IQR 81-88). Patients who underwent segmentectomy had a median of one additional day DAOH90 compared with those who underwent lobectomy (87 vs 86 days, *P* = .049) (**Table 2** and **Figure 2**).

**Figure 3.**
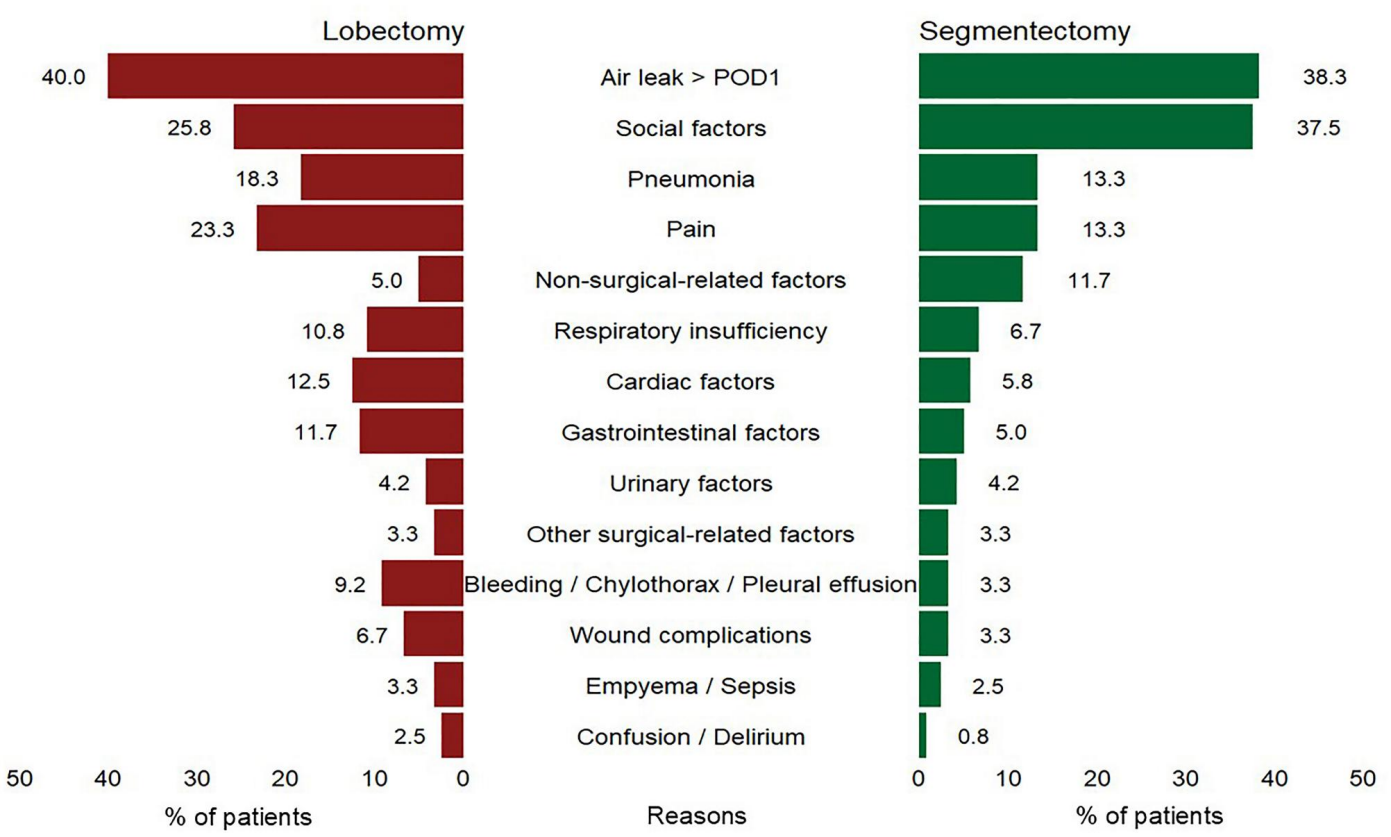
Overall Reasons for Reduced First 90 Days Alive and Out of Hospital (DAOH). Air leak is defined as persistence of the initial chest drain > 1 day, or the occurrence of pneumothorax or subcutaneous emphysema requiring chest drain insertion or hospitalization. POD: postoperative day.

**Figure 4.**
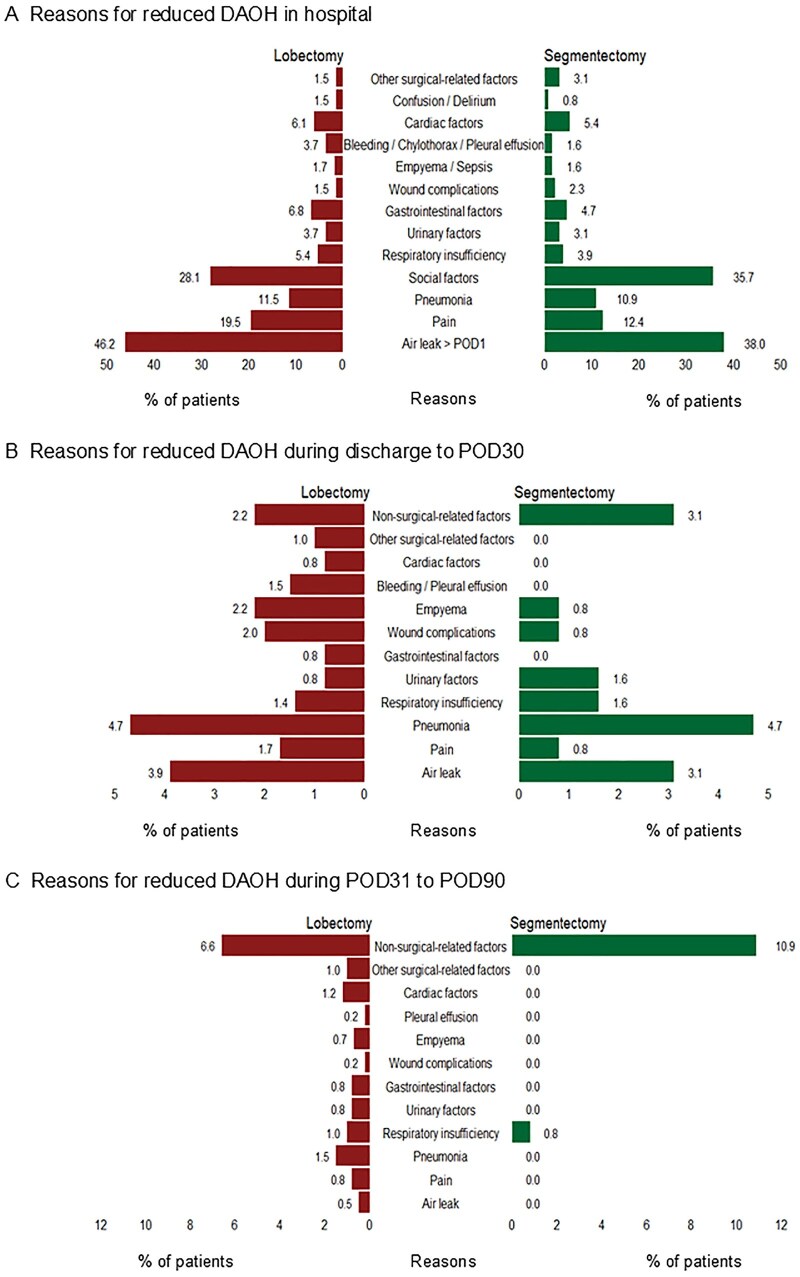
Specific Reasons for Reduced Days Alive and Out of Hospital (DAOH) during Hospitalization, Discharge to Postoperative Day (POD) 30, and POD31-90. Air leak is defined as persistence of the initial chest drain > 1 day, or the occurrence of pneumothorax or subcutaneous emphysema requiring chest drain insertion or hospitalization. POD: postoperative day.

Among contributors to reduced DAOH90 after segmentectomy and lobectomy across the entire 90-day postoperative period, air leak > 1 day was the most frequent (38.3% vs 40.0%), followed by social factors (37.5% vs 25.8%), pneumonia (13.3% vs 18.3%), and pain (13.3% vs 23.3%). Non-surgery-related medical factors were more predominant after segmentectomy (11.7%), whereas cardiac factors (12.5%), gastrointestinal factors (11.7%), and respiratory insufficiency (10.8%) were more common after lobectomy (**Figure 3**). The causes of reduced DAOH90 during the index hospitalization in both groups were consistent with the overall 90-day pattern. However, between discharge and POD30, pneumonia, as the most frequent cause, occurred at the same rate (4.7%) in both groups. From POD31 to POD90, non-surgery-related medical factors (10.9% vs 6.6%) became the predominant contributors, particularly in the segmentectomy group (**Figure 4**).

In the multivariable analysis, higher body mass index (odds (OR) 0.98, 95% confidence interval (CI) 0.93–0.99) and higher FEV_1_%pre (OR 0.99, 95% CI, 0.98–1.00) were associated with a reduced risk of shorter DAOH90, while cardiac comorbidity (OR 1.56, 95% CI, 1.04–2.36) and lobectomy (vs segmentectomy, OR 1.44, 95% CI, 1.01–2.17) increased the risk shorter DAOH90 (**Table 3**).

**Table 3.** Predictors for Shorter DAOH 90 (≤86 Days)

Characteristic	Univariable analysis			Multivariable analysis		
	OR	95% CI	*P*-value	OR	95% CI	*P*-value
Age, per 1-year increase	1.02	1.00, 1.04	**.039**	1.01	1.00, 1.03	.141
Male (vs female)	1.44	1.06, 1.96	**.018**	1.29	0.94, 1.78	.121
BMI, per 0.1 kg/m^2^ increase	0.97	0.94, 1.00	**.021**	0.96	0.93, 0.99	**.005**
Smoke status (vs never)						
Former smoker	1.16	0.73, 1.86	.533	0.95	0.58, 1.57	.844
Current smoker	1.62	0.98, 2.65	.059	1.19	0.70, 2.03	.517
FEV_1_%pre, per 1% increase	0.99	0.98, 0.99	**<.001**	0.99	0.98, 1.00	**.014**
ASA (vs I-II)						
III	1.15	0.79, 1.65	.471			
IV	1.79	0.67, 4.80	.249			
Diabetes	1.91	1.17, 3.12	**.010**	1.69	1.00, 2.86	.050
Hypertension	1.02	0.76, 1.37	.908			
Pulmonary comorbidity	1.67	1.19, 2.36	**.003**	1.30	0.88, 1.90	.184
Cardiac comorbidity	1.84	1.25, 2.70	**.002**	1.56	1.04, 2.36	**.033**
Stroke	0.64	0.35, 1.16	.143			
CCI, per 1 score increase	1.11	1.02, 1.22	**.017**	1.06	0.96, 1.16	.268
Clinical stage IA2 (vs IA1)	0.90	0.65, 1.25	.534			
Location of lesion (vs LLL)						
LUL	0.86	0.53, 1.38	.532			
RLL	1.26	0.88, 1.82	.212			
RUL	1.17	0.75, 1.81	.492			
Lobectomy (vs segmentectomy)	1.26	0.86, 1.84	.243	1.44	1.01, 2.17	**.048**

Bold *P*-value indicates statistical significance.

Abbreviations: BMI: body mass index; CCI: Charlson Comorbidity Index; CI: confidence interval; FEV1%pre: percentage of predicted forced expiratory volume in 1 second; LLL: left lower lobe; LUL: left upper lobe; OR: odds ratio; RLL: right lower lobe; RUL: right upper lobe.

In the sensitivity analysis, 104 matched pairs were generated after PSM with balanced preoperative characteristics (**Table S5** and **Figure S1**). Intraoperative and postoperative outcomes were presented in **Table S6**. The median DAOH90 after segmentectomy was 87 days (IQR 84-88), which was significantly longer than after lobectomy (86 days, IQR 78-88, *P* = .018) (**Figure S2**). The distribution and causes of reduced DAOH90 were similar to those observed in the entire cohort (**Figures S3**  **and**  **S4**).

## DISCCUSION

This study is the first to provide DAOH90 after ERAS VATS segmentectomy. Comparisons with our previous ERAS VATS lobectomy-specific DAOH study demonstrated consistent DAOH90 values and similar patterns of DAOH reduction across postoperative intervals,^10^ thereby reinforcing the reliability of outcomes after lobectomy in the present analysis and offering a robust reference for interpreting the segmentectomy results. Importantly, DAOH90 after segmentectomy was greater than after lobectomy, adding to the evidence in favour of segmentectomy for early-stage NSCLC. Also, these findings highlight detailed shortcomings in perioperative care following segmentectomy, which may be useful to future ERAS strategies.

Data from the Italian VATS Group demonstrated a median LOS of 6.6 days for segmentectomy and 7.2 days for lobectomy, showing no statistical difference.^7^ But analyses from a national Japanese database (7 vs 8 days),^8^ a multicentre Swiss study (6 vs 7 days),^9^ and a Spanish VATS group study (4.8 vs 6.6 days)^14^ reported significantly shorter LOS after VATS segmentectomy compared with lobectomy, consistent with our observations. When considering these actual data, our findings remain favourable. However, it should be noted that the readmission rates in our study were slightly higher than those reported by others, particularly in the segmentectomy group.^7^^,^^15^ In contrast, the 90-day mortality in both groups was lower than that reported in the CALGB trial (lobar resection 1.7%, sublobar resection 1.1%) and the Italian VATS trial (lobectomy 2.0%, segmentectomy 1.4%),^4^^,^^7^ consistently confirming that lobectomy was associated with relatively higher 90-days mortality than segmentectomy, although the difference did not reach statistical significance. Interestingly, perioperative mortality in both groups remained higher than that reported in the Japanese studies (≤0.3% for both).^5^^,^^8^

Overall, VATS segmentectomy may offer perioperative advantages over lobectomy within over 15 years of ERAS implementation at our centre,^10^^,^^11^ as the 1-day difference in DAOH was driven by LOS rather than post-discharge outcomes. But its clinical significance remains uncertain and requires further validation. This study provides important insights, as the causes of reduced DAOH highlight the marginal effects of surgery, whereby unintended events and complications may offset the expected benefits.

Since higher-grade CDC complications occurred more frequently after lobectomy than segmentectomy in this study, this likely contributed to prolonged hospitalization. Strategies to optimize perioperative care for ERAS VATS lobectomy have been addressed in a previous study.^10^ In the present analysis, our focus was on segmentectomy. Management of the intersegmental plane is unique to segmentectomy. At our centre, 3D reconstruction and intraoperative indocyanine green guidance are routinely used to delineate anatomical boundaries and ensure R0 resection while minimizing air leak. Other reported techniques, such as pleural edge closure and coverage with polyglycolic acid mesh and fibrin glue, are not routinely applied. Furthermore, to minimize social factors contributing to prolonged LOS after segmentectomy, preoperative education is essential, as discharge after ERAS VATS segmentectomy is often accelerated, like the situation observed with ERAS VATS wedge resection.^16^ In addition, for further optimizing pain management, early mobilization remains critical.^17^ Plus, structured post-discharge management programs may provide additional benefit in reducing non-surgery-related medical causes of prolonged hospitalization. Last but not least, in high-risk patients with independent predictors of inferior DAOH, prehabilitation and tailored perioperative strategies should be emphasized as key directions for future research and clinical optimization.

This study has several limitations. First, the retrospective design may introduce selection and reporting bias. Although propensity score matching was used to reduce baseline imbalance, subjective outcomes such as social factors and pain are difficult to assess objectively, and surgeon-dependent procedure selection may result in residual confounding by indication. All segmentectomies were performed by highly experienced surgeons and were preferentially applied to small lesions (<2 cm) or patients with limited physiological reserve, with simple resections (eg, culmenectomy, lingulectomy, S6) performed more frequently than technically demanding segmentectomies (eg, S9 or S10). This selection may have reduced postoperative complications, particularly air leak, but limits the ability to determine whether outcomes of complex segmentectomy are comparable to lobectomy. A similar selection bias has been reported in another study,^18^ potentially restricting generalizability to less experienced settings and underscoring the need for prospective validation. Second, no formal power calculation was conducted prior to the study. Based on the available sample size, with a significance level of 0.05, an assumed mean difference in DAOH of 1 day between the segmentectomy and lobectomy groups, and an effect size of 0.5, the calculated statistical power was 0.93, slightly above the conventional threshold of 0.80. Nonetheless, the power for logistic regression analyses may have been insufficient. Moreover, our variable selection strategy may be suboptimal, as pre-screening based solely on univariable significance could exclude clinically relevant predictors that might demonstrate independent associations in a multivariable context. Third, according to the definition of air leak used in this study, the incidence may have been overestimated.

## CONCLUSION

Patients undergoing enhanced recovery thoracoscopic segmentectomy had a median of 87 days alive and out of the hospital within the first 90 postoperative days, which was higher than that observed after lobectomy in this cohort. Postoperative complications were more frequent after lobectomy than after segmentectomy. These findings suggest that segmentectomy, in well-selected patients with clinical stage IA1-2 NSCLC, may provide longer DAOH and fewer postoperative complications compared with lobectomy. Additionally, future optimization of ERAS strategies should focus on reducing prolonged air leak, addressing social factors, and preventing pneumonia and pain.

## Supplementary Material

ivag043_Supplementary_Data

## Data Availability

The full setup raw data are available upon request.
