# Supplementary material for: Thoracoscopic Segmentectomy Within an Enhanced Recovery Pathway Improves Days Alive and Out of Hospital Compared with Lobectomy
Source: Interdiscip Cardiovasc Thorac Surg. 2026 Feb 6;41(2):ivag043. doi: 10.1093/icvts/ivag043 (PMC12906232; doi:10.1093/icvts/ivag043)

| **Supplementary Table 1. The definition of variables in this study.** | |
| --- | --- |
| **Variables** | **Definition** |
| **Age** | Defined as the patient’s age at the time of surgery. |
| **Sex** | Recorded as male or female. |
| **BMI** | Calculated as weight in kilograms divided by height in meters squared (kg/m²). |
| **Smoke status** | Defined as follows: patients who reported smoking up to the day of the preoperative interview were classified as current smokers; those who had quit prior to that date were classified as former smokers; and patients who had never smoked were classified as never smokers. |
| **FEV_1_%pre** | Preoperative value from lung function examination. |
| **ASA classification** | Defined as the preoperative physical status, classified into I-IV based on the evaluation by anesthesiologists. |
| **Diabetes** | According to the International Classification of Diseases, 11th Revision (ICD-11). |
| **Hypertension** | According to ICD-11. |
| **Pulmonary comorbidity** | Defined as pulmonary disease (e.g., COPD, asthma, interstitial lung disease) diagnosed according to ICD-11. |
| **Cardiac comorbidity** | Defined as cardiovascular disease (e.g., coronary artery disease, heart failure, atrial fibrillation) diagnosed according to ICD-11. |
| **Stroke** | According to ICD-11. |
| **CCI** | Defined as the weighted index score of comorbid conditions according to the Charlson Comorbidity Index. |
| **Clinical stage** | According to the 8th edition of the TNM classification. |
| **Location of lesion** | Recorded according to the lobar location of the tumour lesion. |
| **Surgical duration** | Defined as the time from the start to the end of surgery, recorded by the operating surgeon. |
| **Blood loss** | Defined as the total volume of blood loss recorded during surgery. |
| **LOS** | Calculated as the number of nights spent in hospital after surgery. |
| **Duration of chest drainage** | Calculated as the number of nights with a chest drain. |
| **Postoperative complications** | According to ICD-11. |
| **Air leak** | Defined as persistence of the initial chest drain for more than one day, or the occurrence of pneumothorax or subcutaneous emphysema requiring chest drain insertion or hospitalization. |
| **Pain** | Defined as patient-reported pain requiring documentation by the clinician and corresponding treatment. |
| **Social factors** | Recorded when medical records indicated that the patient wished to be discharged the following day, or when discharge was delayed due to the late hour. |
| **Respiratory insufficiency** | Included respiratory failure, oxygen dependency, or dyspnoea. |
| **Urinary factors** | Included urinary tract infection, urinary retention, renal failure, or other dysfunctions. |
| **Gastrointestinal factors** | Included postoperative nausea or vomiting, diarrhea, constipation, gastrointestinal bleeding, or other dysfunctions. |
| **Cardiac factors** | Included atrial fibrillation, heart failure, cardiac arrest, or other cardiovascular diseases. |
| **Non-surgical-related medical factors** | Defined as comorbidities, new disease or others happened after surgery but not associated with surgery. |
| **CDC** | Defined as the standardized grading system for postoperative complications, ranging from Grade I (minor deviation from normal postoperative course) to Grade V (death of a patient). |
| **Readmission** | Defined as any unplanned hospital admission within 90 days after the index surgery. |
| **Mortality** | Defined as all-cause death occurring within 90 days after the index surgery. |
| **Histology of tumour** | Defined as the pathological type of the resected tumour, classified according to the World Health Organization histological classification of lung tumours. |
| BMI: body mass index, CCI: Charlson Comorbidity Index, CDC: Clavin-Dindo classification, FEV_1_%pre: percentage of predicted forced expiratory volume in 1 second, LOS: length of stay. | |

| **Supplementary Table 2. The distribution of resected segments.** | |
| --- | --- |
| **Segmentectomy** | **n = 129^1^** |
| **LS1+2** | 8 (6.2%) |
| **LS1+2+3** | 38 (29.5%) |
| **LS2** | 3 (2.3%) |
| **LS3** | 3 (2.3%) |
| **LS4+5** | 11 (8.5%) |
| **LS6** | 22 (17.1%) |
| **LS6+9** | 1 (0.8%) |
| **LS7+8** | 1 (0.8%) |
| **LS7+8+9** | 2 (1.6%) |
| **LS7+8+9+10** | 3 (2.3%) |
| **LS8** | 1 (0.8%) |
| **LS8+9+10** | 1 (0.8%) |
| **LS9+10** | 1 (0.8%) |
| **RS1** | 1 (0.8%) |
| **RS2** | 2 (1.6%) |
| **RS3** | 2 (1.6%) |
| **RS6** | 16 (12.4%) |
| **RS6+9** | 1 (0.8%) |
| **RS7** | 1 (0.8%) |
| **RS7+8** | 5 (3.9%) |
| **RS7+8+9+10** | 3 (2.3%) |
| **RS9+10** | 3 (2.3%) |
| ^1^n (%); Median (Interquartile range) | |
| LS: segment on the left side, RS: segment on the right side | |

| **Supplementary Table 3. The details of readmissions between lobectomy and segmentectomy.** | | |
| --- | --- | --- |
| **Characteristic** | **Lobectomy**^1^  n = 591 | **Segmentectomy**^1^  n = 129 |
| **Single readmissions** |  |  |
| 0-30-day | 60 (10.2%) | 10 (7.8%) |
| 31-90-day | 26 (4.4%) | 1 (0.8%) |
| **Multiple readmissions** |  |  |
| 0-30-day | 2 (1.9%) | 2 (1.6%) |
| 31-90-day | 3 (0.5%) | 0 (0.0%) |
| **First readmission from discharge within postoperative 30 days, day** | 5 (2, 12) | 4 (2, 10) |
| **Length of readmitted stay within postoperative 30 days, day** | 5 (2, 10) | 4 (2-6) |
| **First readmission from discharge within postoperative 31-90 days, day** | 45 (35-67) | 33 (33-33) |
| **Length of readmitted stay within postoperative 31-90 days, day** | 5 (2.0-11.0) | 1 (1-1) |
| ^1^Median (Interquartile range); n (%) | | |

| **Supplementary Table 4. Pathological outcomes.** | | | | |
| --- | --- | --- | --- | --- |
| **Variables** | **Overall**^1^  n = 720 | **Lobectomy**^1^  n = 591 | **Segmentectomy**^1^  n = 129 | **p-value**^2^ |
| **Histology of tumour** |  |  |  | **0.003** |
| Adenocarcinoma | 575 (79.9%) | 476 (80.5%) | 99 (76.7%) |  |
| Squamous cell carcinoma | 93 (12.9%) | 81 (13.7%) | 12 (9.3%) |  |
| Others | 52 (7.2%) | 34 (5.8%) | 18 (14.0%) |  |
| ^1^n (%); Median (Interquartile range)  ^2^Fisher's exact test; Pearson's Chi-squared test; Wilcoxon rank sum test  Bold p-value indicates statistical significance. | | | | |

| **Supplementary Table 5. Preoperative characteristics after propensity score matching.** | | | | | |
| --- | --- | --- | --- | --- | --- |
| **Characteristic** | **Overall**^1^  n = 208 | **Lobectomy**^1^  n = 104 | **Segmentectomy**^1^  n = 104 | **SMD** | **p-value**^2^ |
| **Age (year)** | 70 (64, 75) | 69 (63, 75) | 71 (66, 76) | -0.03 | 0.167 |
| **Male** | 73 (35.1%) | 32 (30.8%) | 41 (39.4%) | -0.03 | 0.191 |
| **BMI (kg/m^2^)** | 26 (23, 29) | 26 (22, 29) | 25 (23, 28) | 0.06 | 0.587 |
| **Smoke status** |  |  |  | 0.02 | 0.490 |
| Never smoking | 20 (9.6%) | 10 (9.6%) | 10 (9.6%) |  |  |
| Former smoker | 114 (54.8%) | 53 (51.0%) | 61 (58.7%) |  |  |
| Current smoker | 74 (35.6%) | 41 (39.4%) | 33 (31.7%) |  |  |
| **FEV_1_%pre** | 80 (69, 97) | 82 (72, 98) | 80 (66, 95) | -0.00 | 0.198 |
| **ASA** |  |  |  | -0.03 | 0.492 |
| I-II | 35 (16.8%) | 16 (15.4%) | 19 (18.3%) |  |  |
| III | 164 (78.8%) | 85 (81.7%) | 79 (76.0%) |  |  |
| IV | 9 (4.3%) | 3 (2.9%) | 6 (5.8%) |  |  |
| **Diabetes** | 27 (13.0%) | 15 (14.4%) | 12 (11.5%) | 0.04 | 0.536 |
| **Hypertension** | 92 (44.2%) | 48 (46.2%) | 44 (42.3%) | 0.00 | 0.577 |
| **Pulmonary comorbidity** | 49 (23.6%) | 20 (19.2%) | 29 (27.9%) | -0.06 | 0.141 |
| **Cardiac comorbidity** | 33 (15.9%) | 18 (17.3%) | 15 (14.4%) | 0.05 | 0.569 |
| **Stroke** | 18 (8.7%) | 7 (6.7%) | 11 (10.6%) | -0.07 | 0.324 |
| **CCI** | 2 (1, 3) | 2 (1, 3) | 2 (1, 3) | -0.06 | 0.148 |
| **Clinical stage** |  |  |  | 0.05 | 0.672 |
| cIA1 | 85 (40.9%) | 41 (39.4%) | 44 (42.3%) |  |  |
| cIA2 | 123 (59.1%) | 63 (60.6%) | 60 (57.7%) |  |  |
| **Location of lesion** |  |  |  | -0.10 | >0.999 |
| LLL | 52 (25.0%) | 26 (25.0%) | 26 (25.0%) |  |  |
| LUL | 100 (48.1%) | 50 (48.1%) | 50 (48.1%) |  |  |
| RLL | 46 (22.1%) | 23 (22.1%) | 23 (22.1%) |  |  |
| RUL | 10 (4.8%) | 5 (4.8%) | 5 (4.8%) |  |  |
| ^1^Median (Interquartile range); n (%)  ^2^Wilcoxon rank sum test; Pearson's Chi-squared test; Fisher's exact test  BMI: body mass index; CCI: Charlson Comorbidity Index; FEV1%pre: percentage of predicted forced expiratory volume in 1 second; LLL: left lower lobe; LUL: left upper lobe; RLL: right lower lobe; RUL: right upper lobe; SMD: standardized mean difference | | | | | |

| **Supplementary Table 6. Intraoperative, postoperative and pathological characteristics after propensity score matching.** | | | | | | |
| --- | --- | --- | --- | --- | --- | --- |
| **Variables** | **Overall**^1^  n = 208 | **Lobectomy**^1^  n = 104 | **Segmentectomy**^1^  n = 104 | **ARF** | **95% CI** | **p-value**^2^ |
| **Surgical duration (min)** | 100 (90, 115) | 95 (80, 115) | 102 (92, 117) | -6.29 | -13.12 to -1.55 | **0.016** |
| **Blood loss (ml)** | 20 (10, 50) | 20 (10, 50) | 20 (10, 40) | 18.07 | 0.08 to 36.06 | 0.182 |
| **Length of stay (day)** | 3 (2, 5) | 3 (2, 6) | 2 (2, 4) | 1.89 | -0.86 to 4.63 | 0.215 |
| **Duration of chest drainage (day)** | 1 (1, 3) | 2 (1, 3) | 1 (1, 2) | 0.01 | -0.89 to 0.91 | 0.061 |
| **Postoperative complications in hospital** |  |  |  |  |  |  |
| Overall | 80 (38.5%) | 45 (43.3%) | 35 (33.7%) | 10.10 | 4.09 to 32.10 | 0.154 |
| Highest CDC 1 | 35 (16.8%) | 18 (17.3%) | 17 (16.3%) |  |  |  |
| Highest CDC 2 | 22 (10.6%) | 12 (11.5%) | 10 (9.6%) |  |  |  |
| Highest CDC 3 | 19 (9.1%) | 11 (10.6%) | 8 (7.7%) |  |  |  |
| Highest CDC 4 | 3 (1.4%) | 3 (2.9%) | 0 (0.0%) |  |  |  |
| Highest CDC 5 | 1 (0.5%) | 1 (1.0%) | 0 (0.0%) |  |  |  |
| **Postoperative complications during discharge to POD30** |  |  |  |  |  |  |
| Overall | 27 (13.0%) | 19 (18.3%) | 8 (7.7%) | 8.57 | 1.64 to 18.78 | **0.023** |
| Highest CDC 1 | 4 (1.9%) | 3 (2.9%) | 1 (1.0%) |  |  |  |
| Highest CDC 2 | 6 (2.9%) | 4 (3.8%) | 2 (1.9%) |  |  |  |
| Highest CDC 3 | 16 (7.7%) | 12 (11.5%) | 4 (3.8%) |  |  |  |
| Highest CDC 4 | 1 (0.5%) | 0 (0.0%) | 1 (1.0%) |  |  |  |
| Highest CDC 5 | 0 (0.0%) | 0 (0.0%) | 0 (0.0%) |  |  |  |
| **Postoperative complications during POD31 to POD90** |  |  |  |  |  |  |
| Overall | 8 (3.8%) | 7 (6.7%) | 1 (1.0%) | 5.71 | 0.32 to 11.11 | 0.065 |
| Highest CDC 1 | 3 (1.4%) | 2 (1.9%) | 1 (1.0%) |  |  |  |
| Highest CDC 2 | 4 (1.9%) | 4 (3.8%) | 0 (0.0%) |  |  |  |
| Highest CDC 3 | 1 (0.5%) | 1 (1.0%) | 0 (0.0%) |  |  |  |
| Highest CDC 4 | 0 (0.0%) | 0 (0.0%) | 0 (0.0%) |  |  |  |
| Highest CDC 5 | 0 (0.0%) | 0 (0.0%) | 0 (0.0%) |  |  |  |
| **90-days readmission** | 30.0 (14.4%) | 21.0 (20.2%) | 9.0 (8.7%) | 7.61 | 2.47 to 17.71 | **0.018** |
| **90-days mortality** | 2 (1.0%) | 2 (1.9%) | 0 (0.0%) | 0.95 | -1.86 to 3.76 | 0.498 |
| **90 days alive and out of hospital (day)** | 87 (82, 88) | 86 (78, 88) | 87 (84, 88) | -2.23 | -4.86 to -1.04 | **0.018** |
| **Histology of tumour** |  |  |  |  |  | 0.248 |
| Adenocarcinoma | 156 (75.0%) | 78 (75.0%) | 78 (75.0%) |  |  |  |
| Squamous cell carcinoma | 28 (13.5%) | 17 (16.3%) | 11 (10.6%) |  |  |  |
| Others | 24 (11.5%) | 9 (8.7%) | 15 (14.4%) |  |  |  |
| ^1^n (%); Median (Interquartile range)  ^2^Fisher's exact test; Pearson's Chi-squared test; Wilcoxon rank sum test  Bold p-value indicates statistical significance.  ARF: absolute risk difference; CDC: Clavin-Dindo classification; CI: confidence interval; POD: postoperative day | | | | | | |

**Supplementary Figure 1. Standardized Mean Differences Before and After Propensity Score Matching.**

**
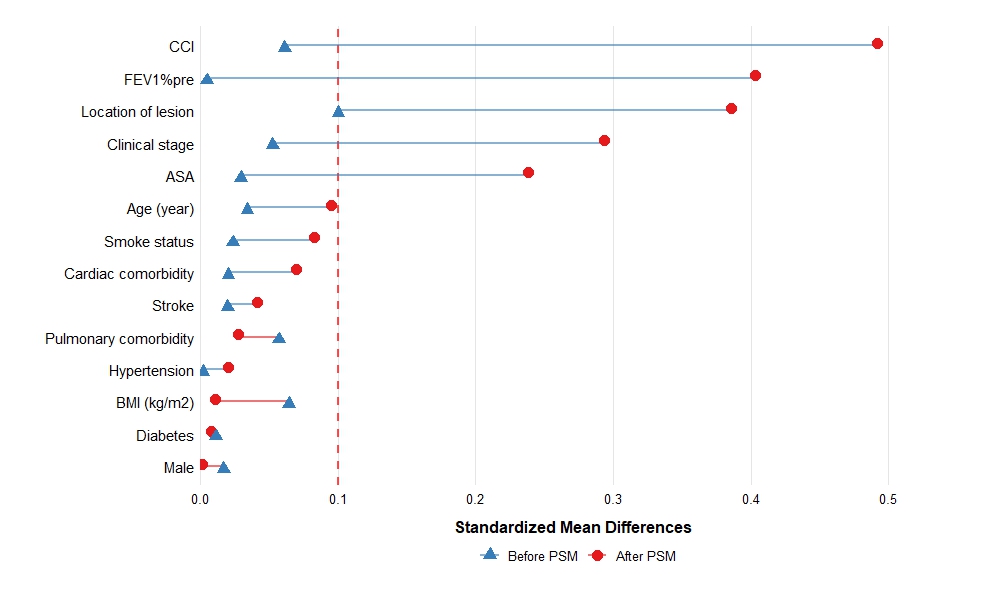
**

**Supplementary Figure 2. Distribution of the first 90 days alive and out of hospital (DAOH) after enhanced recovery thoracoscopic lobectomy and segmentectomy for clinical stage IA1-2 non-small cell lung cancer after propensity score matching (PSM).**

**
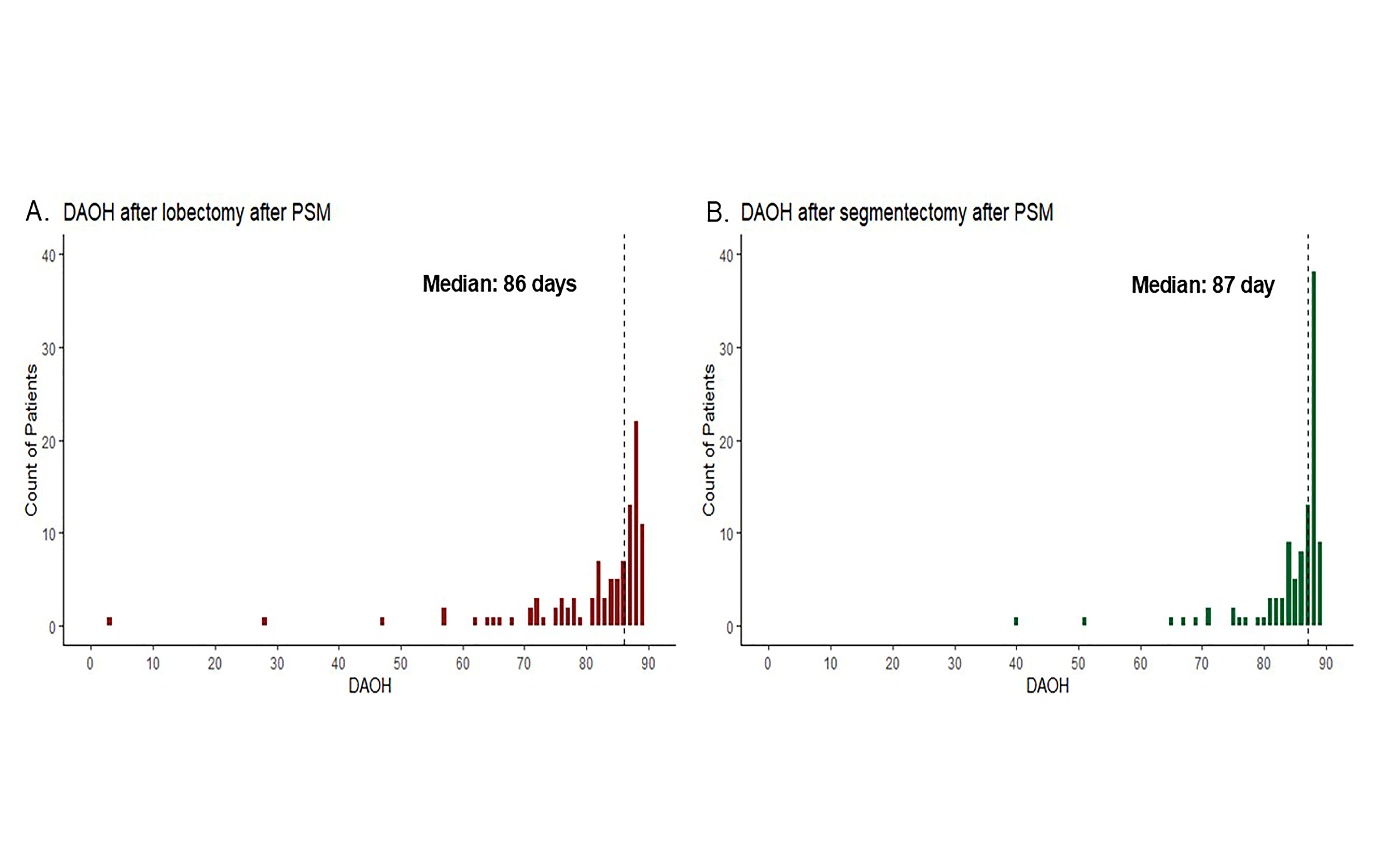
**

**Supplementary Figure 3. Overall reasons for reduced the first 90 days alive and out of hospital (DAOH) after propensity score matching.**


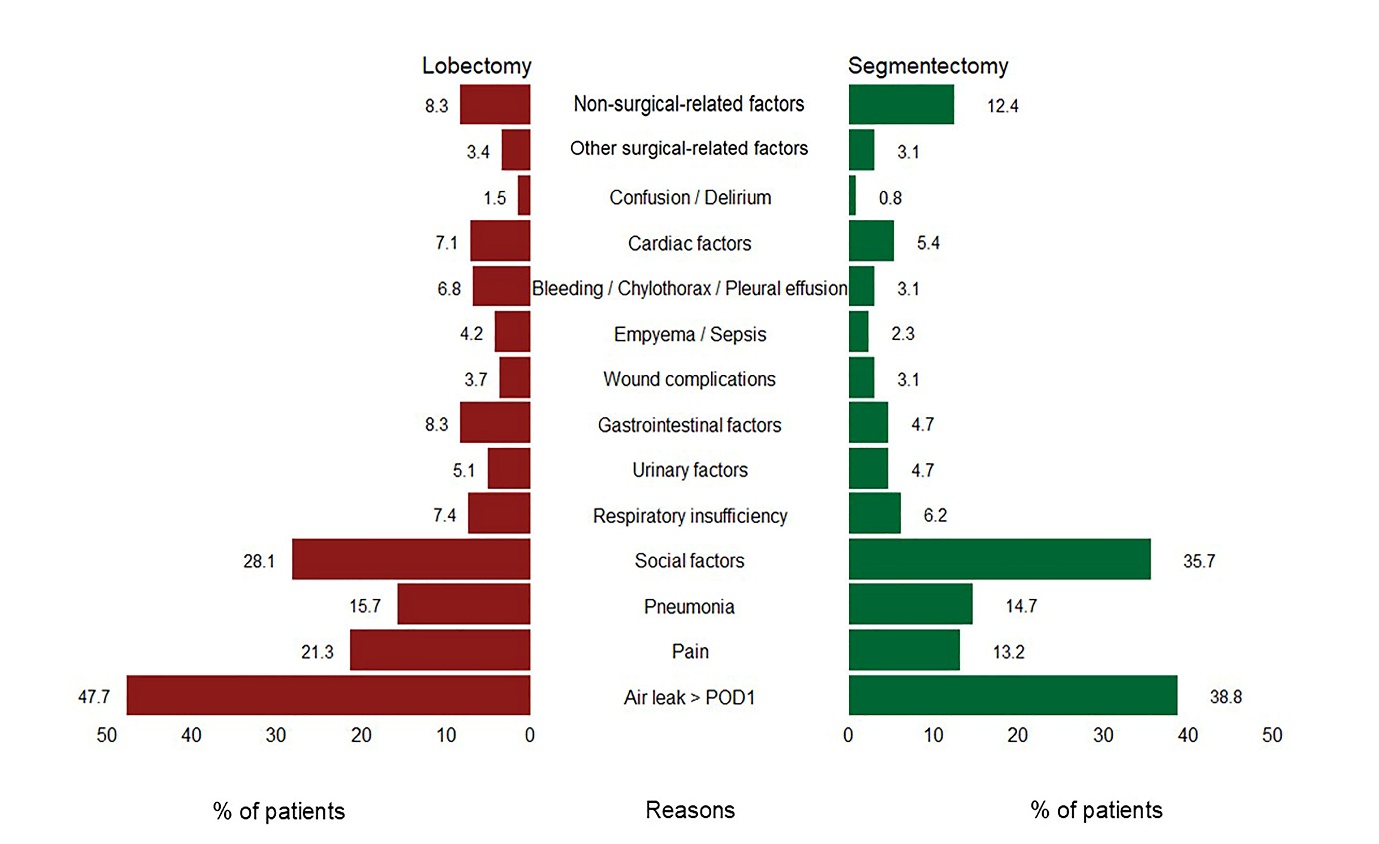


POD: postoperative day

**Supplementary Figure 4. Specific reasons for reduced days alive and out of hospital (DAOH) during hospitalization, discharge to postoperative day (POD) 30, and POD31-90 after propensity score matching (PSM).**


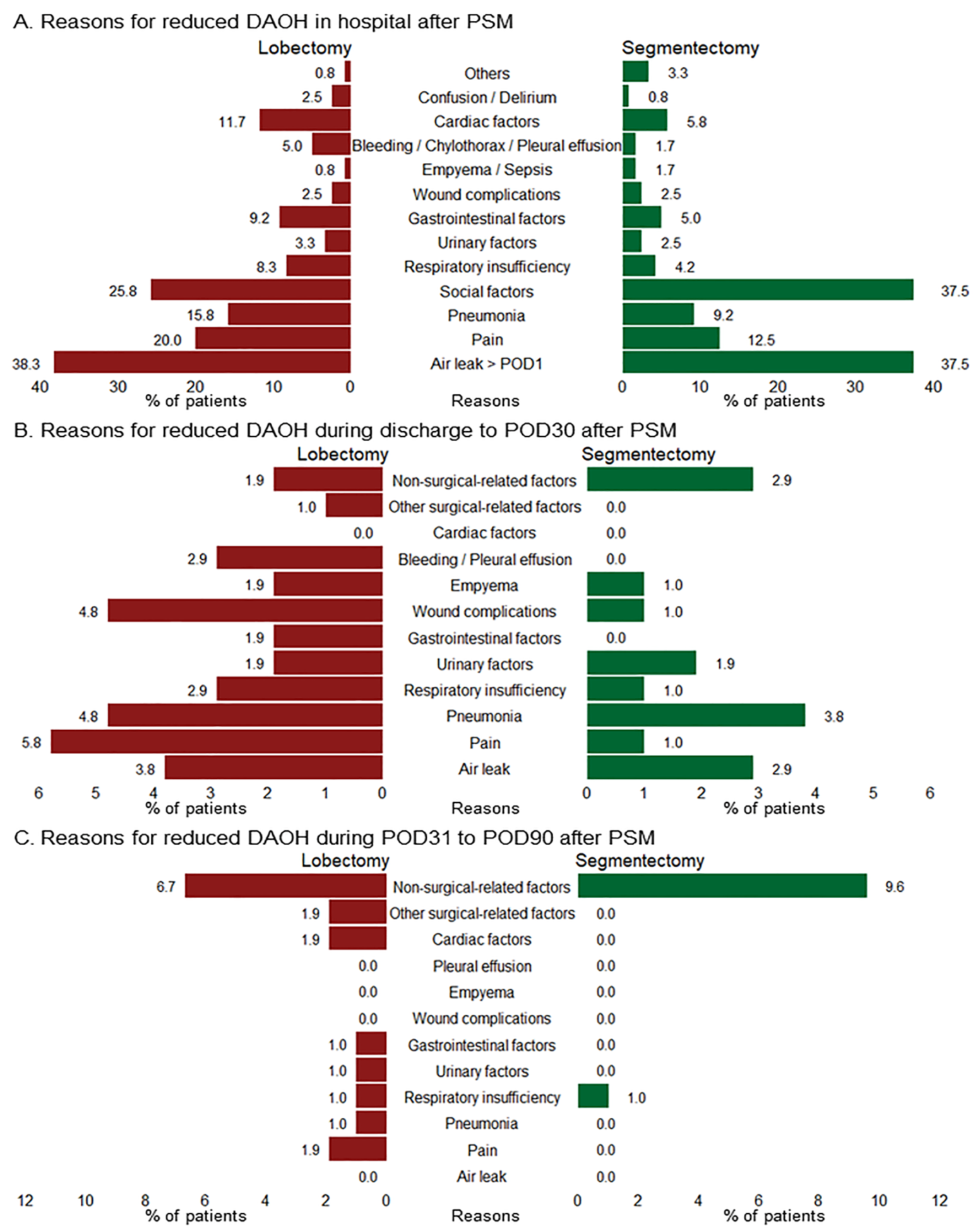

Supplement: ivag043_Supplementary_Data [file ivag043_supplementary_data.docx]
